# Chemical markers for the quality control of herbal medicines: an overview

**DOI:** 10.1186/1749-8546-3-7

**Published:** 2008-06-28

**Authors:** Songlin Li, Quanbin Han, Chunfeng Qiao, Jingzheng Song, Chuen Lung Cheng, Hongxi Xu

**Affiliations:** 1 Chinese Medicine Laboratory, Hong Kong Jockey Club Institute of Chinese Medicine, Hong Kong SAR, PR China

## Abstract

Selection of chemical markers is crucial for the quality control of herbal medicines, including authentication of genuine species, harvesting the best quality raw materials, evaluation of post-harvesting handling, assessment of intermediates and finished products, and detection of harmful or toxic ingredients. Ideal chemical markers should be the therapeutic components of herbal medicines. However, for most herbal medicines, the therapeutic components have not been fully elucidated or easily monitored. Bioactive, characteristic, main, synergistic, correlative, toxic and general components may be selected. This article reviews the effective use of chemical markers in the quality control of herbal medicines including the selection criteria considering the roles and physicochemical factors which may affect the effective use of chemical markers.

## Background

Herbal medicines, also known as botanical medicines or phytomedicines, refer to the medicinal products of plant roots, leaves, barks, seeds, berries or flowers that can be used to promote health and treat diseases. Medicinal use of plants has a long history worldwide. According to the World Health Organization (WHO), traditional herbal preparations account for 30–50% of the total medicinal consumption in China [1]. There have always been concerns about the inconsistent composition of herbal medicines and occasional cases of intoxication by adulterants and/or toxic components. Quality control of herbal medicines aims to ensure their consistency, safety and efficacy.

Chemical fingerprinting has been demonstrated to be a powerful technique for the quality control of herbal medicines. A chemical fingerprint is a unique pattern that indicates the presence of multiple chemical markers within a sample.

The European Medicines Agency (EMEA) defines chemical markers as chemically defined constituents or groups of constituents of a herbal medicinal product which are of interest for quality control purposes regardless whether they possess any therapeutic activity [2]. Ideally, chemical markers should be unique components that contribute to the therapeutic effects of a herbal medicine. As only a small number of chemical compounds were shown to have clear pharmacological actions, other chemical components are also used as markers. The quantity of a chemical marker can be an indicator of the quality of a herbal medicine.

The overall quality of a herbal medicine may be affected by many factors, including seasonal changes, harvesting time, cultivation sites, post-harvesting processing, adulterants or substitutes of raw materials, and procedures in extraction and preparation. From harvesting to manufacturing, chemical markers play a crucial role in evaluating the quality of herbal medicines. Moreover, the study of chemical markers is applicable to many research areas, including authentication of genuine species, search for new resources or substitutes of raw materials, optimization of extraction and purification methods, structure elucidation and purity determination. Systematic investigations using chemical markers may lead to discoveries and development of new drugs.

In this review, we summarise selection criteria for chemical markers and how chemical markers are used to evaluate the quality of herbal medicines.

### Selection of chemical markers

A total of 282 chemical markers are listed in the Chinese Pharmacopoeia (2005 edition) for the quality control of Chinese herbal medicines [3]. As discussed in the monographs of the American Herbal Pharmacopoeia (AHP), the use of single or multiple chemical markers was important to quality control [4]. Scientists and regulatory agencies have paid attention to the selection of chemical markers in quality control. The EMEA categorises chemical markers into analytical markers and active markers [2]. According to the definition by the EMEA, analytical markers are the constituents or groups of constituents that serve solely for analytical purposes, whereas active markers are the constituents or groups of constituents that contribute to therapeutic activities. There are other classifications of chemical markers. For example, Srinivasan proposed the following four categories: active principles, active markers, analytical markers and negative markers [5]. Active principles possess known clinical activities; active markers contribute to clinical efficacy; analytical markers have no clinical or pharmacological activities; negative markers demonstrate allergenic or toxic properties. All markers may contribute to the evaluation, standardisation and safety assessment of herbal medicines. Lin *et al. *expanded Srinivasan's classification into seven categories, namely active principles, active markers, group markers, chemical fingerprints, analytical markers, 'phantom' markers and negative markers [6]. Group chemical markers have similar chemical structures and/or physical properties. The pharmacological activities of individual components are not necessarily known. Polysaccharides are classified under this category. This type of markers is not necessarily specific and can be easily masked by other components especially in proprietary products. 'Phantom' markers are constituents that have known pharmacological activities; however, they can be undetectable in some herbal medicines due to low quantities. Special care should be taken when 'phantom' markers were selected as chemical markers for quality control [6]. While group chemical markers have a lower resolving power in qualitative analysis, chemical fingerprinting cannot provide adequate quantitative information.

In this paper, we suggest a new classification of eight categories of chemical markers, namely (1) therapeutic components, (2) bioactive components, (3) synergistic components, (4) characteristic components, (5) main components, (6) correlative components, (7) toxic components, and (8) general components used with fingerprint spectrum. These eight categories are defined and discussed in the subsequent sections.

#### Therapeutic components

Therapeutic components possess direct therapeutic effects of a herbal medicine. They may be used as chemical markers for both qualitative and quantitative assessments.

Originated from the bulbs of *Fritillaria *species (family Liliaceae), *Bulbus Fritillariae *(*Beimu*) is commonly prescribed as an antitussive and expectorant in Chinese medicine practice. Five different *Bulbus Fritillariae *derived from nine *Fritillaria *species are documented in the Chinese Pharmacopoeia [3]. Isosteroidal alkaloids of *Bulbus Fritillariae*, including verticine, verticinone and imperialine, were identified as the major therapeutic components that account for the antitussive effect [7-9]. Therefore, isosteroidal alkaloids were selected as the chemical markers for the quality assessment of *Bulbus Fritillariae *using a series of chromatographic techniques such as pre-column derivatizing gas chromatography – flame ionization detection (GC-FID), direct GC-FID, gas chromatography – mass spectrometry (GC-MS), pre-column derivatizing high-performance liquid chromatography – ultraviolet detection (HPLC-UV), high-performance liquid chromatography – evaporative light scattering detection (HPLC-ELSD) and high-performance liquid chromatography – mass spectrometry (HPLC-MS) methods [10].

Artemisinin from *Herba Artemisiae Annuae *(*Qinghao*) is another example of therapeutic component. *Herba Artemisiae Annuae *is well known for its potent anti-malarial activity [11]. Artemisinin inhibits *Plasmodium falciparum *and *Plasmodium vivax*, two pathogens that cause malaria [12,13]. Artemisinin is now used as a chemical marker in HPLC-ELSD [14], GC-FID [15] and GC-MS [15,16] for assessing the quality of the plant (parts and whole) at various stages [15], including the green and dead leaves of the plant [16].

#### Bioactive components

Bioactive components are structurally different chemicals within a herbal medicine; while individual components may not have direct therapeutic effects, the combination of their bioactivities does contribute to the therapeutic effects. Bioactive components may be used as chemical markers for qualitative and quantitative assessment.

According to Chinese medicine theories, *Radix Astragali *(*Huangqi*), derived from the roots of *Astragalus membranaceus *(Fish.) Bge. or *A. membranaceus *var. *mongholicus *(Bge.) Hsiao, is used to reinforce *qi*. Isoflavonoids, saponins and polysaccharides of *Radix Astragali *showed pharmacological actions in immune and circulatory systems, which were consistent with the Chinese medicine indications [17]. These bioactive components, including isoflavonoids and saponins, were used simultaneously in the evaluation of the quality of *Radix Astragali *[18-20].

#### Synergistic components

Synergistic components do not contribute to the therapeutic effects or related bioactivities directly. However, they act synergistically to reinforce the bioactivities of other components, thereby modulating the therapeutic effects of the herbal medicine. Synergistic components may be used as chemical markers for qualitative and quantitative assessment.

The products of St John's wort (*Hypericum perforatum *L.) are popular for treating mild depression [21]. Butterweck *et al. *reviewed the research progress on the phytochemistry and pharmacology of St John's wort [22,23]. Naphthodianthrone, hypericin, and hyperforin (a phloroglucinol derivative) were identified as the major components that contribute to the pharmacological activities of St John's wort. Rutin, a ubiquitous flavonoid of natural products, demonstrated synergistic antidepressant actions in St John's wort [24]. In a forced swimming test on rats, extracts of St John's wort with various chemical profiles were tested, among which the extract containing about 3% of rutin showed positive effects, whereas the extracts containing less than 3% of rutin were inactive. The extracts became active when the level of rutin was increased to about 3%. However, rutin alone did not show any effects under the same conditions [24]. These results suggest that chemicals in St John's wort work synergistically to achieve the antidepressant effects. Therefore, naphthodianthrones, phloroglucinols and flavonoids may be used as chemical markers for the quality control of St John's wort [25-29].

#### Characteristic components

While characteristic components may contribute to the therapeutic effects, they must be specific and/or unique ingredients of a herbal medicine.

Terpene lactones in the leaves of *Ginkgo biloba *L. (*Yinxing*) exemplify characteristic components. EGb 761, a standardized leave extract of *Ginkgo biloba *is a well defined product for the treatment of cardiovascular diseases, memory loss and cognitive disorders associated with age-related dementia [30]. Flavonoids and terpene lactones are responsible for the medicinal effects of EGb 761 [31]. Flavonoids, terpene lactones including ginkgolides A, B and C, and bilobalide are chemical markers for the quality control of *Ginkgo biloba *leave extracts [31-34]. EGb 761 contains 6% of terpene lactones (2.8–3.4% of ginkgolides A, B and C, and 2.6–3.2% of bilobalide) and 24% of flavone glycosides. Aglycons are primarily quercetin, kaempferol and isorhamnetin.

Valerenic acids, the characteristic components of valerian derived from the roots of *Valeriana officinalis *L., have sedative effects and improve sleep quality [35,36]. Valerenic acids are used as chemical markers to evaluate the quality of valerian preparations although their sedative effects have not been fully elucidated [37]. These chemical markers are also used for studying *in vitro *release of coated and uncoated tablets [38] and stability test for valerian ground materials and extracts [39].

#### Main components

Main components are the most abundant in a herbal medicine (or significantly more abundant than other components). They are not characteristic components and their bioactivities may not be known. Main components may be used for both qualitative and quantitative analysis of herbal medicines especially for differentiation and stability evaluation.

Four well-known Chinese herbal medicines derived from the genus *Panax*, namely (1) *Radix et Rhizoma Ginseng *(*Renshen*), (2) *Radix et Rhizoma Ginseng Rubra *(*Hongshen*), (3) *Radix Panacis Quinquefolii *(*Xiyangshen*) and (4) *Radix et Rhizoma Notoginseng *(*Sanqi*) [39], contain triterpenoid saponins including ginsenoside Rg1, Re, Rb1 and notoginsenoside R1 as their main components [40-42]. Through qualitative and quantitative comparison of the saponin profiles [43-45], these four herbs can be differentiated from one another [42,46].

*Herba Epimedii *(*Yinyanghuo*), derived from the aerial parts of *Epimedium brevicornum *Maxim., *E. sagittatum *(Sieb. et Zucc.) Maxim., *E. pubescens *Maxim., *E. wushanense *T. S. Ying or *E. koreanum *Nakai, has been traditionally used to reinforce kidney-yang (*shenyang*), strengthen tendons and bones, and relieve rheumatic conditions [47]. Flavonoids including epimedin A, B, C and icariin are the main components of *Herba Epimedii *[48]. A 24-month randomised, double-blinded and placebo-controlled clinical study showed that flavonoids from *Herba Epimedii *exerted beneficial effects on preventing osteopenia in late post-menopausal women [49]. According to the Chinese Pharmacopoeia (2005 edition), total flavonoids and icariin are used as chemical markers for *Herba Epimedii *[47]. In a recent study, Chen *et al*. developed an HPLC method to simultaneously quantify up to 15 flavonoids, of which epimedin A, B, C and icariin were selected as chemical markers for the quality assessment of the *Epimedium *species documented in the Chinese Pharmacopoeia (2005 edition) [50].

#### Correlative components

Correlative components in herbal medicines have close relationship with one another. For example, these components may be the precursors, products or metabolites of a chemical or enzymatic reaction. Correlative components can be used as chemical markers to evaluate the quality of herbal medicines originated from different geographical regions and stored for different periods of time.

**Figure 1:**
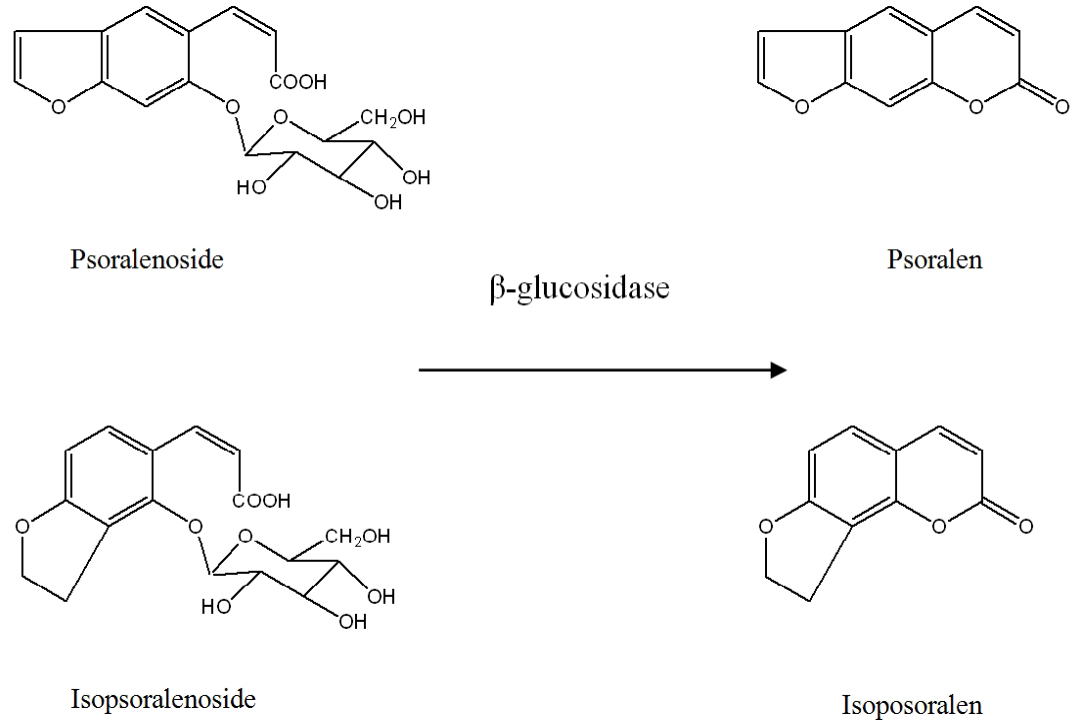
Psoralen, isopsoralen, psoralenoside, isopsoralenoside and their correlation in *Psoralea corylifolia *L.

According to the Chinese Pharmacopoeia (2005 edition), only psoralen and isopsoralen (Figure 1) are used as chemical markers for assessing the quality of *Fructus Psoraleae *(*Buguzhi*) [51]. Recently, our group identified glycosides, psoralenoside and isopsoralenoside (Figure 1) found them useful as the chemical markers for *Fructus Psoraleae *[52]. The levels of psoralen and isopsoralen are inversely correlated to the level of glycosides psoralenoside and isopsoralenoside [53]. When extracted with 50% methanol, samples had high levels of psoralen and isopsoralen and a minute amount of psoralenoside and isopsoralenoside. When moistened with 100% methanol, dried and extracted with 50% methanol, samples contained all four components. Psoralen and isopsoralen may be the enzymatic reaction products of psoralenoside and isopsoralenoside respectively. After incubation of a psoralenoside and isopsoralenoside solution with β-glucosidase at 36°C for 24 hours, the amount of psoralen and isopsoralen became detected. Psoralenoside and isopsoralenoside, therefore, may be used as chemical markers for the quality control of *Fructus Psoraleae *[53].

#### Toxic components

Traditional Chinese medicine literature and modern toxicological studies documented some toxic components of medicinal herbs. For instance, aristolochic acids (AAs) and pyrrolizidine alkaloids (PAs) may cause nephrotoxicity and heptotoxicity respectively [54,55].

The use of three herbal medicines that contain AAs, namely *Radix Aristolochiae Fangchi *(*Guangfangji*), *Caulis Aristolochiae Manshuriensis *(*Guanmutong*) and *Radix Aristolochiae *(*Qingmuxiang*), have been prohibited in China since 2004 [56]. These three herbs were traditionally used to relieve pain and treat arthritis. *Radix et Rhizoma Asari *(*Xixin*) was traditionally sourced from the whole plants of *Asarum heterotropoides *Fr. Schmidt var. *mandshurcum *(maxim.) Kitag., *A. sieboldii *Miq. var. *seoulense *Nakai or *A. seiboldii *Miq. Its medicinal use has now been officially limited to the roots and rhizomes because the roots and rhizomes contain a much lower level of AAs than the aerial parts [57]. AAs are now used as markers to control nephrotoxic herbs and proprietary herbal products [58-61].

There are over 6,000 plant species containing PAs which can cause hepatic veno-occlusive disease [62]. There have been various PA restriction guidelines issued by government bodies and organisations. According to the WHO guidelines issued in 1989, the lowest intake rate of toxic PAs that may cause veno-occlusive disease in human is 15 μg/kg/day [63]. In 1993, the American Herbal Products Association (AHPA) alerted its members to restrict the use of comfrey, a herbal medicine that contains PAs for external applications. In 2001, the Food and Drug Administration (FDA) of the United States recalled comfrey from all dietary supplements [64]. In 2007, the Medicines and Healthcare Products Regulatory Agency (MHRA) of the United Kingdom advised all herbal interest groups to withdraw all unlicensed proprietary products that may contain hepatotoxic PAs from *Senecio *species [65]. PAs are thus markers for detection of hepatotoxic components in herbs [66-72].

#### General components coupled with 'fingerprints'

General components are common and specific components present in a particular species, genus or family. These components may be used with 'fingerprints' for quality control purposes.

Lobetyolin, a polyacetylene compound, is used as a marker for *Radix Codonopsis *(*Dangshen*) in thin-layer chromatography (TLC). *Radix Codonopsis *is derived from the roots of three *Codonopsis *species, namely *Codonopsis pilosula *(Franch.) Nannf., *C. pilosula *Nannf. var. *modesta *(Nannf.) L. T. Shen or *C. tangshen *Oliv. [73]. Our study showed that other five *Codonopsis *species that are common substitutes of *Radix Codonopsis *also contain lobetyolin. They are *C*.*tubulosa *Kom., *C*.*subglobosa *W. W. Smith, *C*. *clematidea *(Schynek) C. B. Cl., *C*.*canescens *Nannf. and *C*.*lanceolata *(Sieb. et Zucc.) Trautv. Moreover, the roots of *Campanumoea javanica *Bl. and *Platycodon grandiflorum *(Jacq.) A. DC. (familyCampanulaceae), which are easily confused with *Radix Codonopsis*, also contained lobetyolin. Therefore, lobetyolin may be used as a general chemical marker coupled with HPLC-UV 'fingerprints' to differentiate *Radix Codonopsis *from its substitutes and adulterants [74].

As a chemical component may have more than one attribute, a component may belong to multiple categories. For example, ginkgolides A, B and C, and bilobalide are not only characteristic components, but also bioactive components of *Ginkgo biloba*. Ginsenoside Rg1, Re and Rb1 are both main and bioactive components of *Panax ginseng*. Categories of chemical markers are summarised in Table 1.

**Table 1:** Quality control interests and disadvantages of chemical markers

Category	Quality control interests	Disadvantages
Therapeutic components	Indicating efficacy	Not always obtained
Toxic components	Safety assurance	Need extensive toxicological studies
Bioactive components	May indicate efficacy	Not always indicate the overall quality
Main components	Stability and consistency test	Not always indicate the overall quality
Characteristic components	Qualitative identification	Not always obtained
Synergistic components	Revealing synergistic actions of multi-components	Need extensive pharmacological studies
Correlative components	Prediction of storage period, extraction methods and sometimes collection site etc.	Need extensive phytochemical studies
General components used together with fingerprint spectrum	Indicating overall quality when used together with fingerprint spectrum	Mass data analysis

### Applications of chemical markers

In this section, we describe cases to exemplify how chemical markers are used to evaluate the quality of herbal medicines in manufacturing, and as potential lead compounds for new drug development.

#### Identification of adulterants

**Figure 2:**
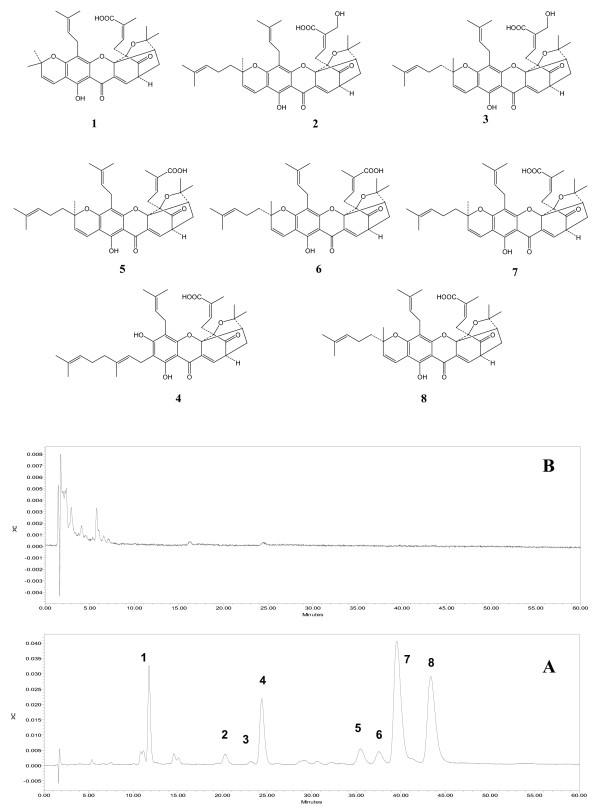
**HPLC chromatograms of gamboges (A) and its adulterant (B)**. **1**, Morellic acid; **2**, *R*-30-hydroxygambogic acid; **3**, S-30-hydroxygambogic acid; **4**, Gambogenic acid; **5**, *R*-isogambogic acid; **6**, *S*-isogambogic acid; **7**, *R*-gambogic acid; **8**, *S*-gambogic acid.

Derived from the resin of *Garcinia hanburyi *Hook f. (family Guttiferae), gamboges (*Tenghuang*) has been used in China to treat scabies, tinea and malignant boil [75], and in Thailand to treat infected wounds, pain and oedema [76]. Characteristic polyprenylated caged xanthones including gambogic acid, gambogenic acid were isolated as the main and bioactive components of gamboges [77]. In our previous study, an adulterant of gamboges was differentiated from the authentic sample by an HPLC-UV method using eight caged xanthones as chemical markers. The chromatogram of gamboges had all eight compounds, while the adulterant showed none of them (Figure 2).

#### Differentiation of herbal medicines with multiple sources

*Radix Stemonae *(*Baibu*) is a traditional antitussive and insecticidal herbal medicine derived from the roots of three *Stemonae *species, namely *Stemona tuberosa *Lour, *S. sessilifolia *(Mig.) Mig. and *S. japonica *Mig. [78]. The *Stemona *alkaloids were pharmacologically proven to be responsible for the antitussive and insecticidal effects of *Radix Stemonae *[79-83]. In our studies, we observed that the chemical profiles of these three *Stemona *species varied greatly. Croomine-typealkaloids such as croomine were detected in all three species, while protostemonine-type alkloids such as protostemonine and maistemonine were detected in *S. japonica *and *S. sessilifolia*. Moreover, stichoneurine-type alkaloids such as stemoninine, neotuberostemonine and tuberostemonine were only found in *S. tuberosa*. *Stemona *alkaloids may be used as markers to discriminate the three *Stemona *species [84,85].

#### Determination of the best harvesting time

*Rhizoma Chuanxiong *(*Chuanxiong*) is one of the traditional Chinese medicinal herbs frequently used to treat cerebro- and cardio-vascular diseases. Various chemical compounds have been isolated and identified from *Rhizoma Chuanxiong*, including ferulic acid, senkyunolide I, senkyunolide H, senkyunolide A, coniferyl ferulate, Z-ligustilide, 3-butylidenephthalide, riligustilide and levistolide A [86-92]. These chemicals have multiple biological activities which may contribute to the therapeutic effects of the herb [93-99]. Thus, major bioactive components senkyunolide A, coniferyl ferulate, Z-ligustilide, ferulic acid, 3-butylidenephthalide, riligustilide and levistolide A may be used as markers to select the best harvesting time. A previous study using these markers suggested that the best harvesting time for *Rhizoma Chuanxiong *is from mid April to late May [100].

#### Confirmation of collection sites

In our studies on the chemistry and antitussive activities of *Radix Stemonae *[101-103], four chemical profiles of *S. tuberosa *of different geographic sources were characterised using croomine, stemoninine, neotuberostemonine or tuberostemonine as markers [102]. Moreover, the total alkaloid of *S. tuberosa *exhibited various levels of antitussive activities in a citric acid-induced guinea pig cough model [82]. Croomine, stemoninine, neotuberostemonine and tuberostemonine all possess significant antitussive activities, however, croomine (croomine type) act on the central nervous system pathway, whereas the other three alkaloids (stichoneurine type) acted on the peripheral pathway of cough reflex [82]. In terms of safety, those containing stichoneurine-type alkaloids are more suitable *Radix Stemonae *sources than those containing croomine as the major component. Croomine, stemoninine, neotuberostemonine, and tuberostemonine may be used as markers to confirm the collection sites for *S. tuberosa *(e.g. Shizhu and Erbian in Sichuan province, Masupo and Baoshan in Yunnan province, Shanglin in Guangxi province or Yudu in Jiangxi province, China) which contains higher levels of stemoninine, neotuberostemonine or tuberostemonine, and a low level of croomine [102].

#### Assessment of processing methods

In general practice, most herbs must be processed to reduce toxicity. For example, *Radix Aconiti *(*Chuanwu*) derived from the root of *Aconitum carmichaeli *Debx [104], is a well known toxic and potent herbal medicine. Cases of intoxication and even death were reported in China and Japan [105-107]. The herb is processed by boiling in water for 4–6 hours or steaming for 6–8 hours [108]. The toxic components of this herb are diester-diterpene *Aconitum *alkaloids, such as aconitine, mesaconitine and hypaconitine. When processed, these alkaloids hydrolyse into their respective analogues collectively known as monoester alkaloids [109]. Monoester alkaloids are much less toxic than diester alkaloids [110]. These six *Aconitum *alkaloids may be used to evaluate *Radix Aconiti *[111].

#### Quality evaluation of herbal parts

Traditionally, *Radix Astragali *is graded according to its diameter, length and physical appearance. Isoflavonoids and saponins were recognised as the major bioactive components attributed to the therapeutic effects of *Radix Astragali*. These two types of components were used to evaluate the quality of *Radix Astragali *in our study, in which 25 samples of *Radix Astragali *were collected from four cultivating regions in China [112]. The contents of 11 main isoflavonoids and three major astragalosides were analysed. Contrary to the traditional notion, thin roots contained more astragalosides than thick ones. Moreover, the content of astragalosides in the bark were over 74-fold higher than that in the xylem. There was no difference in isoflavonoid content between the thin and thick roots, or the bark and the xylem. These results suggest that the thin root *Radix Astragali *is of better quality [112].

#### Identification and quantitative determination of proprietary products

*Qingfu Guanjie Shu *(QGS, also known as JCICM-6) capsule is a proprietary product to treat rheumatoid arthritis. QGS has significant suppressive effects on arthritic [113] and acute inflammation in animal models [114]. The formula of QGS is composed of five anti-inflammatory and anti-arthritic herbs, namely *Caulis Sinomenii*, *Radix Paeoniae Alba*, *Cortex Moutan*, *Rhizoma Curcumae Longae *and *Radix Aconiti Lateralis Preparata*. Sinomenine, paeoniflorin, paeonol, cucurmin and hypaconitine are the major constituents of the five herbs respectively, all of which have significant *in vivo *and *in vitro *effects including anti-inflammation, analgesia, anti-arthritis and immunosuppression [115-118]. Thus, HPLC methods were developed with these five chemicals as markers in the manufacturing process of QGS [111,119]. Immediately after production, three batches of QGS products were examined and no remarkable variations in terms of the five chemicals were found [119].

#### Stability test of proprietary products

Stability test is used to evaluate product quality over time and determine recommended shelf life. The five markers mentioned above were used as indicators to evaluate the product stability of QGS. For example, the accelerated conditional stability test was carried out with four time points in a period of three months in chambers at 40 ± 2°C and 75 ± 5% of humidity. The five markers were quite stable during the period; only paeonol showed a slight decrease of 5% immediately after production [119].

#### Diagnosis of herbal intoxication

Toxic components may be used as chemical markers in screening methods, e.g. rapid diagnosis of acute hidden aconite poisoning in urine samples by HPLC-MS [107]. Five pairs of aconite alkaloids (i.e. aconitine and benzoylaconitine, yunaconitine and deacetyl-yunaconitine, mesaconitine and benzoylmesaconitine, hypaconitine and benzoylhypaconitine, and crasscauline A and deacetyl-crasscauline A) were chosen as markers to develop a LC-MS screening method. The screening method was applied to a clinical investigation of 15 cases of suspected herbal poisoning, of which 11 cases were tested by LC-MS [107].

#### Lead compounds for new drug discovery

The components responsible for the therapeutic effects may be investigated as lead compounds for new drug discovery. Gambogic acid (Figure 2), one of the major caged xanthones of gamboges, is used as a chemical marker for quality control and safety evaluation of gamboges [120-123]. As its cytotoxicity is attributed to cell apoptosis induction [124-128], gambogic acid is a potential lead compound for new anti-cancer drugs [124-126] and has recently been approved by the State Food and Drug Administration of China for clinical trials of cancer treatment [129].

### Problems

Chemical markers are indispensable to quality control of herbal medicines; yet many problems remain to be solved.

#### Shortage of chemical markers

At present, some herbs do not have markers for quality control. According to the Chinese Pharmacopoeia (2005 edition), only 281 out of 551 herbs have one or two chemical markers for quality control. A total of 282 chemical markers have been listed [3] for qualitative or quantitative analysis of herbs. Moreover, many herbal medicines share the same chemical markers for quality control (Table 2).

**Table 2:** Chemical markers shared by various herbal medicines in the Chinese Pharmacopoeia (2005 English version)

Chemical marker	Herb	Page no
Paeoniflorin	*Radix Paeoniae Alba*	222
	*Radix Paeoniae Rubra*	223
Chlorogenic acid	*Flos Lonicerae*	62
	*Folium Eucommiae*	70
	*Flos Chrysanthemi*	57
	*Folium Pyrrosiae*	76
	*Flos Loniceae Japonicae*	62
	*Herba Saussureae Involucratae*	151
Hyperoside	*Folium Crataegi*	69
	*Herba Hyperici Perforati*	139
Rutin	*Folium Mori*	74
	*Flos Sophorae*	65
	*Herba Saussureae Involucratae*	151
Naringin	*Exocarpium Citri Grandis*	52
	*Fructus Aurantii*	83
	*Rhizoma Drynariae*	264
Berberine	*Rhizoma Coptidis*	256
	*Cortex Phellodendron Amuren*	47
	*Cortex Phellodendron Chinensis*	48
	*Caulis Mahoniae*	26
Quercetin	*Herba Euphobiae Humifusa*	137
	*Folium Apocyni Veneti*	67
	*Herba Lysimachiae*	143
Ferulic acid	*Radix Angelicae Sinensis*	191
	*Rhizoma et Radix Ligustici*	266
Scutellarin	*Herba Scutellariae Barbatae*	153
	*Herba Erigerontis*	135
Osthole	*Fructus Cnidii*	90
	*Radix Angenicae Pubescentis*	190
Gentiopicrin	*Radix Gentianae Macrophyllae*	216
	*Radix et Rhizoma Gentianae*	204
Buddleoside	*Flos Buddlejae*	54
	*Flos Chrysanthemi Indici*	58

#### Unqualified purity

Inconsistency in quality is a common prolem among the commercially available chemical markers. The overall quality of chemical markers may be influenced by various physical and chemical factors such as described and exemplified below.

#### Solvents

**Figure 3:**
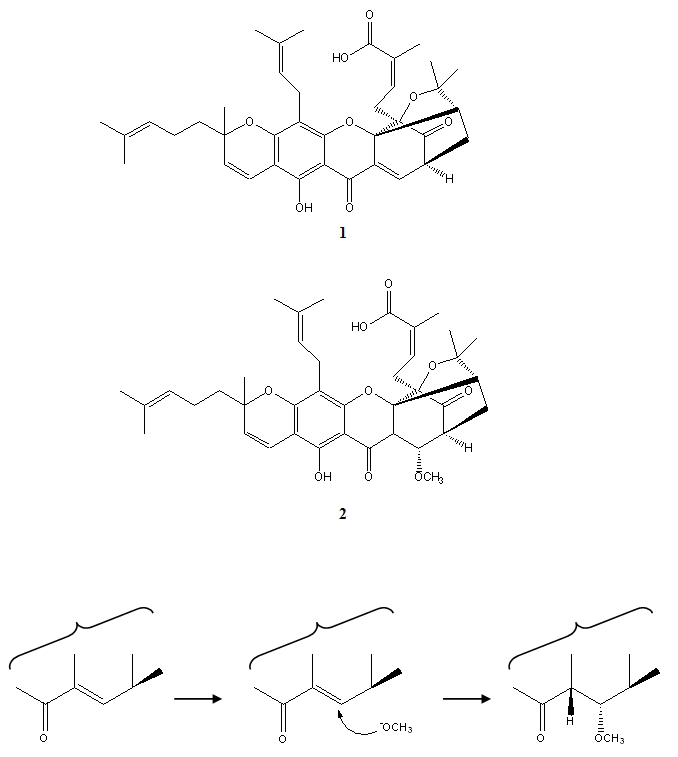
Gambogic acid (**1**) and gambogoic acid (**2**) and the possible derivative schemes.

Gambogic acid is one of the major characteristic caged xanthones in gamboges and is therefore an ideal chemical marker for quality control. However, during the isolation and purification processes, gambogic acid can be transformed by the nucleophilic addition of methanol to the olefinic bond at C-10 when stored in methanol solution at room temperature (Figure 3) [130].

#### Temperature

**Figure 4:**
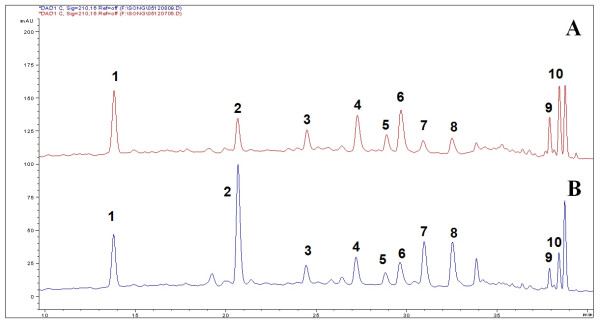
**HPLC chromatograms of *Radix Astragali *with reflux extraction (A) and with microwave assisted extraction (B)**. (1) Calycosin-7-*O*-β-D-glucoside, (2) Calycosin-7-*O*-β-D-glucoside-6"-*O*-malonate, (3) Ononin, (4) (6a*R*,11a*R*)-3-Hydroxy-9,10-dimeth-oxypterocarpan-3-*O*-β-D-glucoside, (5) (3*R*)-7,29-Dihydroxy-39,49-dimethoxy-isoflavan-7-*O*-β-D-glucoside, (6) Calycosin, (7) Formononetin-7-*O*-b-D-glucoside-6"-*O*-malonate, (8) Astrapterocarpanglucoside-6"-*O*-malonate, (9) Formononetin, (10) (6a*R*,11a*R*)-3-Hydroxy-9,10-dimeth-oxypterocarpan.

**Figure 5:**
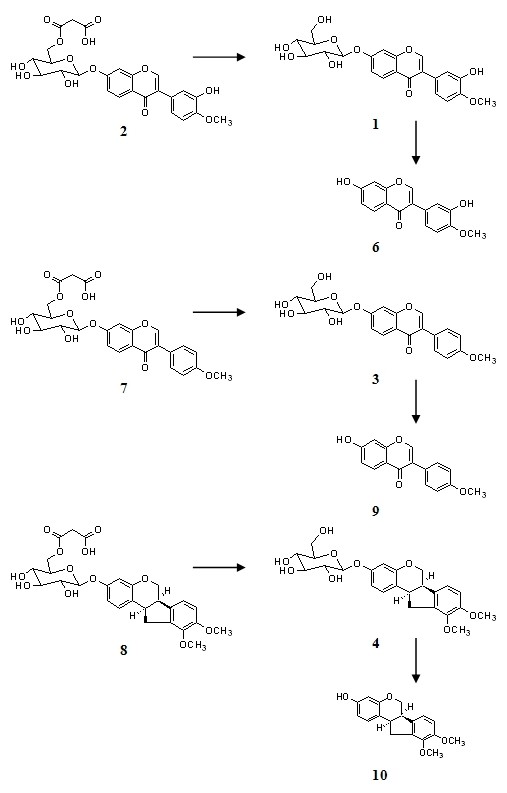
**Possible schemes of conversion of flavonoid glycoside malonates to their related flavonoid glycoside and flavonoid during reflux extraction of *Radix Astragali***. (1) Calycosin-7-*O*-β-D-glucoside, (2) Calycosin-7-*O*-β-D-glucoside-6"-*O*-malonate, (3) Ononin, (4) (6a*R*,11a*R*)-3-Hydroxy-9,10-dimeth-oxypterocarpan-3-*O*-β-D-glucoside, (5) (3*R*)-7,29-Dihydroxy-39,49-dimethoxy-isoflavan-7-*O*-β-D-glucoside, (6) Calycosin, (7) Formononetin-7-*O*-b-D-glucoside-6"-*O*-malonate, (8) Astrapterocarpanglucoside-6"-*O*-malonate, (9) Formononetin, (10) (6a*R*,11a*R*)-3-Hydroxy-9,10-dimeth-oxypterocarpan.

Bioactive components of *Radix Astragali*, isoflavonoids have been used as chemical markers in the quality control of the herb [18-20]. The relative contents of flavonoids varied significantly among samples obtained with different extraction methods (Figure 4). The chemical profiles of samples with microwave assisted extraction, reflux or Soxhlet were compared. Isoflavonoid glycoside malonates were converted into their respective glycosides or flavonoid glycons (Figure 5) during the prolonged conventional extraction procedures with Soxhlet and reflux at higher temperature [18].

#### Light

**Figure 6:**
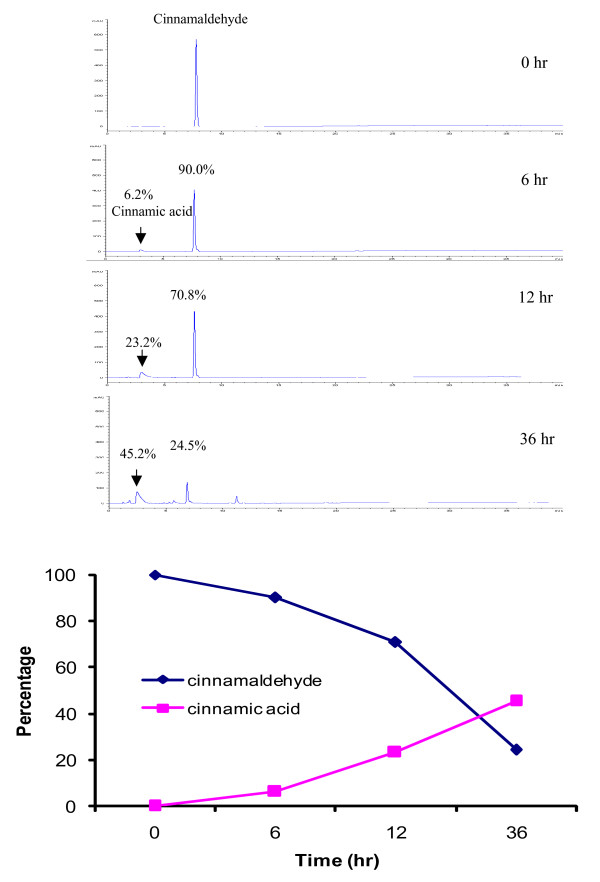
Determination of cinnamaldehyde samples exposed to light in different durations.

**Figure 7:**
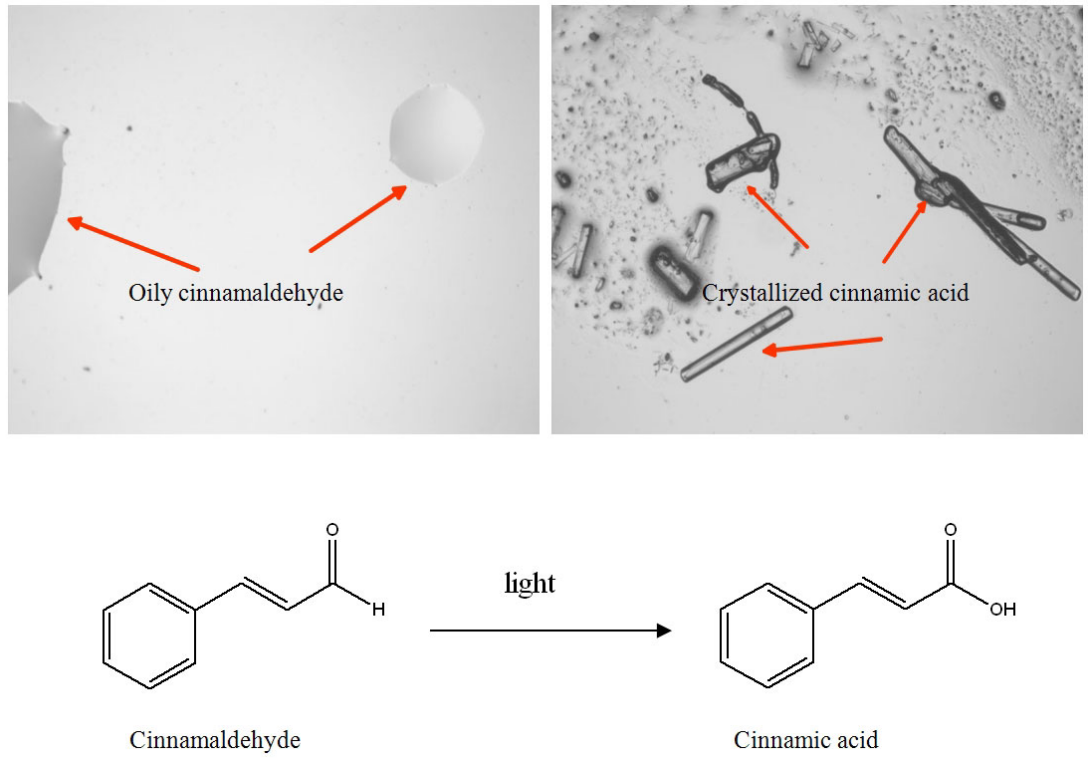
Conversion of cinnamaldehyde to cinnamic acid when exposed to light.

Cinnamaldehyde is the chemical marker for the quantitative evaluation of *Cortex Cinnamomi *[131]. This compound is light-sensitive. When exposed to light at room temperature for six hours, 10% of the content of cinnamaldehyde was lost; and 36 hours later, only 25% was left (Figure 6). Later studies indicated that this compound gradually transformed to crystallized cinnamic acid when exposed to light (Figure 7).

#### Epimeric mixture

Stereoisomers of some phytochemicals often co-exist in nature and are sometimes mistakenly isolated as 'pure' compounds. Most stereoisomers possess very different bioactivities from each other. Gambogic acid, a chemical marker for the gamboges [120,121], has two epimers i.e. 2(*R*)-gambogic acid and 2(*S*)-gambogic acid in nature [77,132]. The epimers have different inhibitory effects on CYP2C9 which is an important enzyme in the liver. The two epimers should be used as separate chemical markers [77]. The two epimers were eluted as a fine peak on a C_18 _column; they can be separated on a C_8 _column under optimized conditions [122,123].

#### Conformations

Spectral complexity of a compound sometimes leads to confusion on its purity. Biflavonoids, for instance, always show substantial spectral complexity at the dimeric level due to hindered rotation between the flavanone and the flavanonol moieties around the C-3/C-8" axis. GB1 (3",4',4"',5,5",7',7"-heptahydroxy-3,8"-biflavanone) is a major biflavanoids of *Garcinia kola *Heckel which is a plant native to Nigeria and Ghana and is often chewed by the locals for tooth cleaning. GB1 is an ideal marker for *G. kola*. The NMR spectra of GB1 obtained at 21°C exhibited two sets of signals, which appeared to be a mixture of two characteristic conformations. When measured at 70°C and 90°C, GB1 showed a single set of signals, the higher the temperature, the faster the hindered rotation between the flavanone and the flavanonol moieties around C-3/C-8" axis. When the rotation was fast enough, the compound existed at a relatively stable conformation, and the spectral complexity at the dimeric level disappeared. When measured at much lower temperatures such as -30°C and -50°C, GB1 showed the same behaviour [133]. We finally confirmed that GB1 was a pure compound [133].

## Conclusion

Quality control of herbal medicines aims to ensure its quality, safety and efficacy. Chemical markers are pivotal in the current practice of quality control. Chemical markers should be used at various stages of the development and manufacturing of a herbal medicine, such as authentication and differentiation of species, collecting and harvesting, quality evaluation, stability assessment, diagnosis of intoxication and discovery of lead compounds. Lack of chemical markers remains a major problem for the quality control of herbal medicines. In many cases, we do not have sufficient chemical and pharmacological data of chemical markers. Furthermore, there are many technical challenges in the production of chemical markers. For example, temperature, light and solvents often cause degradation and/or transformation of purified components; isomers and conformations may also cause confusions of chemical markers.

## Abbreviations

AAs: aristolochic acids; PAs: pyrrolizidine alkaloids; QGS: *Qingfu Guanjie Shu*; TLC: thin layer chromatography; GC-FID: gas chromatography – flame ionization detection; GC-MS: gas chromatography – mass spectrometry; HPLC-UV: high-performance liquid chromatography – ultraviolet detection; HPLC-ELSD: high-performance liquid chromatography – evaporative light scattering detection; HPLC-MS: high-performance liquid chromatography – mass spectrometry

## Competing interests

The authors declare that they have no competing interests.

## Authors' contributions

SL collected references and drafted the manuscript, QH, CQ, JS and CC participated in the discussion and drafting of the manuscript, HX conceived the idea and finalized the manuscript. All authors read and approved the final version of the manuscript.
